# Smart green CQD@SiO_2_ hybrid coated optical fiber manifesting dual versatile absorptive and MIP features towards epinephrine detection†

**DOI:** 10.1039/d2na00687a

**Published:** 2022-12-19

**Authors:** T. Azargoshasb, R. Parvizi, F. Bozorgzadeh, H. Ali Navid, H. Heidari

**Affiliations:** a Department of Laser and Optical Engineering, University of Bonab Bonab 5551761167 Iran; b Department of Physics, College of Sciences, Yasouj University Yasouj 75914-353 Iran roghaieh.parvizi@glasgow.ac.ir; c Physics Department, College of Sciences, Shiraz University Shiraz Iran; d James Watt School of Engineering, University of Glasgow Glasgow G12 8QQ UK

## Abstract

For the first time, in this study, a novel optical fiber biosensor is proposed and developed *via* coating only one smart functional layer of silica-supported carbon dots realizing the concepts of both lossy mode resonance (LMR) and molecularly imprinted polymer (MIP) for epinephrine detection. The carbon quantum dots (CQDs) are prepared using a green synthesis method and then treated with a molecularly imprinted polymer (MIP) strategy. Under ultrasonic irradiation, a SiO_2_ shell was stabilized on the surface of the CQDs to graft and to provide the LMR/MIP functional layer onto the curved optical fiber surface. Accurate structural and morphological characterization confirmed the carbon quantum dot agents and also the SiO_2_ supporting shells on the optical fiber, while spectroscopic analysis confirms the formation of the imprinted polymer and desirable absorbance characteristics. The experimental and numerical sensing studies revealed that the proposed sensing probe allows the rapid adsorption/desorption of epinephrine to the sensing films and highly permeable coating for studying the influence of effective parameters. Under the optimal experimental conditions, the sensitivity of the proposed LMR-based optical fiber sensor is reported to be 0.37 nm μM^−1^ with a correlation coefficient of 0.99. So, sensitive detection of epinephrine at a low concentration can be guaranteed with a 0.72 mM LOD.

## Abstract 2

Smart functional layer of silica-supported carbon dots coated optical fiber biosensor realizing the concepts of both lossy mode resonance (LMR) and molecularly imprinted polymer (MIP) for epinephrine detection.

## Graphical abstract

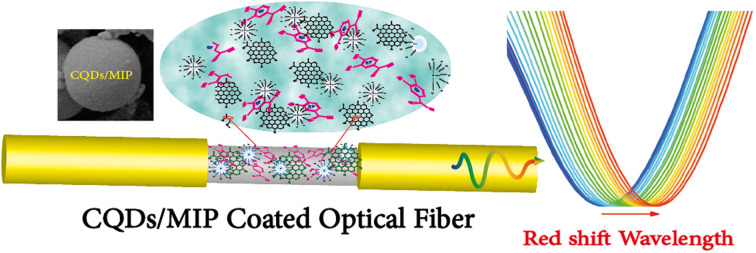

## 1. Introduction

Carbon-based materials offer a wide range of technical uses, especially in photonics and optoelectronics. These substances are characterized by good stability, eco-friendliness, low toxicity, significant thermo-mechanical properties, and a low-cost synthesis process. Recently, carbon-based quantum dots, have attracted significant attention due to their high photo-stability, strong luminescence, and tunable optical properties. In 2004, luminescent carbon quantum dots (CQDs) were accidentally discovered by Xu *et al.* during the purification of single-walled carbon nanotubes through preparative electrophoresis.^1^ The strong and long-lasting luminescence properties of CQDs can be used in sensors for detecting biomolecules,^2^ cellular imaging^3^ and information encryption.^4^ Nowadays, CQDs are being developed as a sustainable alternative to heavy metal-based quantum dots. During the last decade, CQDs have been synthesized using chemical, laser ablation, electrochemical, microwave, and hydrothermal/solvothermal methods.^5^ Since the conventional approaches suffer from complicated economic and environmental issues, considerable efforts have been made to improve green synthesis methods with less harmful precursors.^6^ CQD nanoparticles have several benefits, but they must be functionalized to work. To use CQDs in optical fiber sensors, they must be embedded in a solid matrix to generate thin film structures that preserve their absorptive and analyte-binding capabilities.^7^ In this case, the molecular imprinting technique can be used to improve the selectivity of CQD-based sensors.^8^ Furthermore, light traveling through an optical fiber can interact with its environment to generate evanescent, surface plasmon polariton (SPP), Bloch, lossy/leaky, or guided surface waves.^9^ These waves are sensitive to changes in the medium around the optical fiber, making them ideal for sensing applications. It is well known that the lossy mode resonance (LMR) occurs as a result of a coupling between a guiding mode and a particular lossy mode of the semiconductor thin-layer, which depends on two conditions: a considerable overlap between mode fields and the phase-matching condition.^10^ Several promising fiber optic sensing approaches have been proposed, which benefit from the small size, corrosion resistance, and low-cost fiber optics. For example, the curcumin-based CQDs synthesized using microwave and functionalized with APTES are used to design an innovative fiber-optic dopamine sensor.^11^ Since the detection of target molecules in complex samples is challenging due to the overlapping between the auto-optical properties of some samples and the CQDs' fluorescence emission spectrum,^12^ molecularly imprinted polymers (MIPs) have been exploited along with the CQDs to improve the selectivity, sensitivity, and anti-interference feature of CQD-based sensors.^13^ The core–shell CQDs coupled with MIPs are used to detect cancer biomarkers.^14^ Recently, Arabi *et al.* have studied the green perspectives and strategies of MIPs.^15^ Also, dummy MIPs were prepared based on a green strategy for magnetic solid-phase extraction of acrylamide in biscuit samples.^16^ A novel fluorescent MIP nanosensor based on doped graphene QDs was fabricated for octopamine (OA) detection.^17^ Comprehensive reviews of MIP-based sensors with luminescent carbon dots are given in ref. 13 and 18.

The integration of nano-science and fiber optics has opened up the possibility of developing highly sensitive, selective, and reproducible bio-sensing platforms for real-life applications.^19^ Epinephrine (EP), more commonly known as adrenaline, is a catecholamine neurotransmitter or hormone which plays a vital role in the central nervous system. EP modifies the body's metabolism *via* redistributing blood to the muscles and is responsible for increasing cardiac output and raising the glucose levels and pressure in the blood. Measuring the amount of EP in a patient's fluids is of great importance since the abnormal EP concentrations might relate to specific medical disorders.^21^ For example, it has been recently found that Parkinson's and Alzheimer's diseases, and other neurological disorders are strongly affected by the EP concentration in blood and urine.^22^

In this work, an optical fiber sensor has been developed for the determination of EP based on the LMR/MIP technique. To the best of our knowledge, this is the first experimental demonstration of the CQD-based LMR optical fiber selective sensor. Aligned with the advantages of green chemistry standards, the silica-supported CQDs were successfully prepared using a carbon precursor by an ultrasonic irradiation process. Our approach is low-cost, non-toxic, and eco-friendly since spinach is used as the carbon source and ethanol/methanol as the solvent medium. The as-synthesized CQDs were characterized by HR-TEM, FE-SEM, XRD, FT-IR, and ultraviolet-visible (UV-Vis) spectroscopy and the mechanism of fluorescence emission was investigated.

## 2. Experimental methods

### 2.1 Preparation and characterization of green-CQDs

**Fig. 1:**
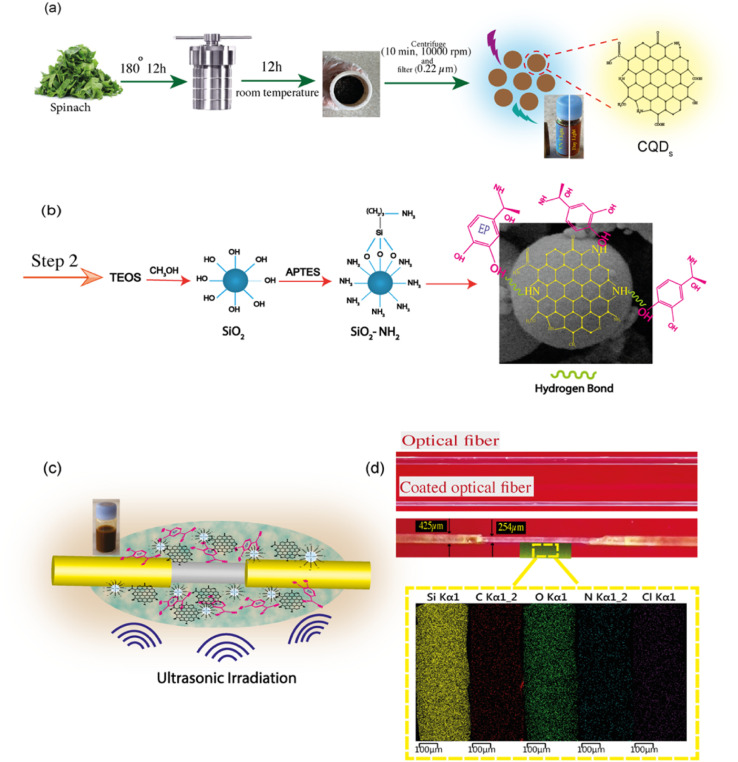
(a) Schematic illustration of the synthesis of carbon dot nanoparticles from spinach; sample viewed under visible and ultraviolet light, (b) the chemical reaction at the core of the optical fiber, (c) immersed optical fiber in the solution under ultrasonic irradiation, (d) image of the surface-coated optical fiber along with the elemental mapping of the carbon dots.

In order to synthesize CQDs *via* a green strategy, spinach leaves (100 g) were used as the carbon-based precursor and were washed carefully with water to remove any dust. Following, the leaves were washed with distilled water and then cut into small pieces. For hydrothermal treatment, a small portion of spinach (20 g) was added to 10 mL of water in a Teflon-lined stainless-steel autoclave with a volume capacity of 50 mL and was heated up to 180 °C and kept for 12 h (see Fig. 1). After the autoclave cooled to room temperature naturally, the brown mixture was centrifuged at 2000 and then 10 000 rpm for 10 min to separate any non-reactive materials from the CQD solution. Then, the resulting product was filtered through a 0.22 μm membrane filter to remove large insoluble carbonaceous particles. The CQD supernatant was collected and stored at 4 °C for further characterization and device fabrication.

### 2.2 Preparation of the CQDs/MIP coated probe

After the etching of the fiber, explained in the ESI,† the SiO_2_ structure was obtained with the copolymerization of APTES, TEOS, and methanol. First, 1.5 mL of TEOS, 0.7 mL of APTES, and 10 mL of methanol were mixed together, and the reaction system was kept under magnetic stirring for 30 min. The prepared solution was mixed with 3 drops of glycine as a nitrogen source and 0.5 mL of diluted HCl until the pH value reached 5–6. The final mixture solution was further stirred for 10 min at room temperature. The as-synthesized CQDs and EP (9 mL of 100 ppm EP solution) were added to the solution. After that, optical fibers were immersed in this solution, and an ultrasonic bath was employed to disperse nanoparticles efficiently. The schematics of the chemical reaction occurring at the core of the optical fiber coated with the carbon dots are illustrated in Fig. 1(b). As a result of the cavitation effect, a homogeneous dispersion is attained for further use in the sensing region of the optical fiber. Finally, to get the MIP layer, the template molecules were removed from the imprinted polymer layer by immersing the probe in methanol and acetic acid (9 : 1) for a required time. This washing time was determined by repeating the cycle sensing measurements for each probe washed at different times. The non-imprinted polymers (NIPs) on the CQDs called CQDs/NIPs were synthesized according to the same procedure, while no EP was added through polymerization. An image of the MIP-coated optical fiber along with the elemental mapping of the CQDs is shown in Fig. 1(d) (see ESI S1† for more details).

## 3. Results and discussion

### 3.1 Characterization of the CQDs

**Fig. 2:**
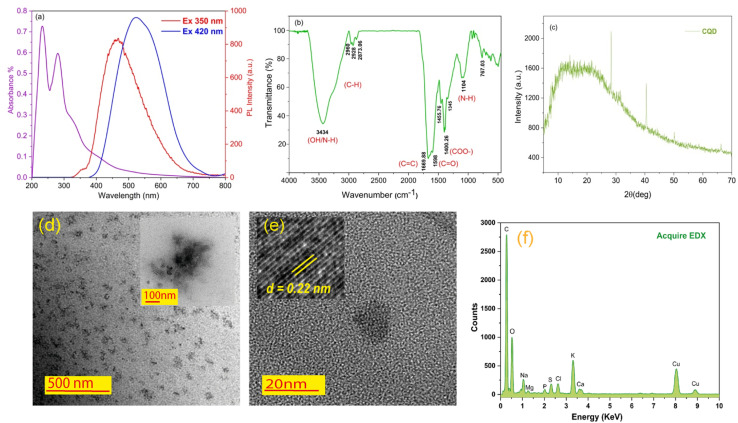
(a) The UV-Vis absorption and PL intensity spectra at different excitation wavelengths of 350 and 420 nm, (b) FT-IR spectrum, (c) XRD pattern, (d) HR-TEM images, (e) magnified HR-TEM and inset showing the lattice fringe distance at 0.22 nm, and (f) EDX elemental mapping of the synthesized carbon dots.

To investigate the optical properties of the synthesized spinach-CQDs, the UV absorbance was characterized by UV-Vis spectroscopy. The UV-Vis absorbance spectrum of CQDs exhibits a strong optical absorption with two distinct sharp peaks in the UV region. The main absorption peak near 233 nm was assigned to the typical π → π* transition of the conjugated C=C bond, and the shoulder peak near 281 nm corresponds to the n → π* electronic transition of C=O.^23,24^ There is no background absorption in the visible area, indicating that no other forms of the CQD nanocomposite are produced during the partial carbonization of precursors.^25^Fig. 2(a) depicts the excitation wavelength dependence of the PL spectrum of the CQD nanocomposite. The excitation spectra were monitored at two excitation wavelengths, 350 nm to 420 nm. It is evident that the PL peak position experiences a red shift upon increasing the excitation wavelength. Furthermore, the PL intensity is lower for the excitation wavelength of 350 nm (red line) than the case for 420 nm (blue line). This excitation-dependent spectrum feature is possibly related to the existence of surface energy trap distribution and different particle sizes.^26^ FT-IR transmittance spectra identify CQD surface functional groups. As shown in Fig. 2(b), the characteristic absorption bands of the –OH stretching vibration mode could be observed at about 3434 cm^−1^. The bands at 2960, 2928, and 2880 cm^−1^ correspond to the C–H stretching mode. In addition, the peaks appearing at 1300 to 1550 cm^−1^ may represent aromatic nitrogen heterocycles.^27^ Also, the peaks at 1669 and 1104 cm^−1^ were assigned to C=C and N–H wagging, respectively.^28^ Therefore, the surface functional groups on the CQDs were predicted to be hydroxyl (–OH), carboxyl (C=O), amide (CONH_2_), and epoxide/ether (C–O–C) groups, which makes the CQDs highly dispersible in water. The XRD pattern of CQDs is shown in Fig. 2(c). The broad peak around 2*θ* = 23° corresponds to the (0 0 2) plane of the carbon material, which signifies the amorphous nature of CQDs.^29^

The morphology and structures of the as-synthesized CQDs were examined by high-resolution transmission electron microscopy (HR-TEM). As shown in Fig. 1(d), a close observation of the spinach-CQDs suggests that CQDs are spherical nanostructures and well dispersed in water, without any aggregation. The size and shape distributions were confirmed as spherical by HR-TEM (extended graphs can be found in ESI S6†). The lattice fringe distance is observed at 0.22 nm, corresponding to the (1 0 0) in-plane lattice of graphite carbon (Fig. 1(e)).^30^ Based on the obtained HR-TEM image, it has been concluded that the average diameter of the synthesized CQDs was about 8 nm. The EDX elemental mapping image of the spinach-CQD nanocomposite is shown in Fig. 2(f), demonstrating that surface-functionalized CQDs were successfully produced (see ESI S1†).

### 3.2 Characterization of the CQDs/MIP nano-composites

**Fig. 3:**
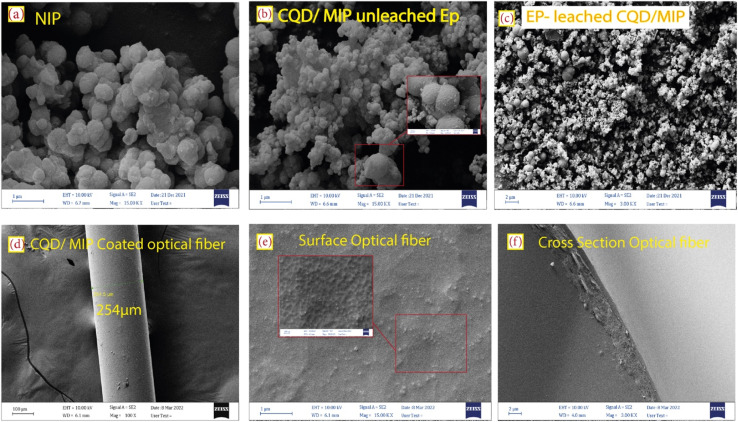
FE-SEM micrographs of (a) NIP, (b) unleached and (c) EP-leached CQDs/MIPs, (d and e) top view and (f) cross-section of the CQDs/MIP coated optical fiber.

Fig. 3(a–f) display the FE-SEM micrographs of the non-imprinted polymer (NIP), unleached CQDs/MIPs, EP-leached CQDs/MIP powder, EP-leached CQDs/MIP coated optical fiber and the cross-section of CQDs/MIPs, respectively. The morphology and diameter of the CQDs/NIP and CQDs/MIP nanocomposites are similar, both nanocomposites are sphere-shaped, and their diameters are less than 100 nm. Also, the porous structure is observed in Fig. 3(b and c).

**Fig. 4:**
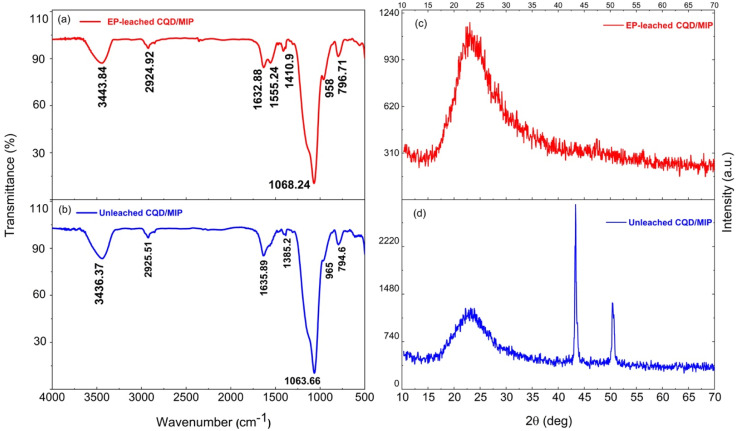
FT-IR spectrum and the XRD pattern of (a) and (c) EP-leached CQDs/MIPs, and (b) and (d) unleached EP CQDs/MIPs.

Fig. 4 shows the surface functional groups on the leached and unleached CQDs/MIPs before EP, which were recognized using FT-IR spectra. Comparing Fig. 4(a and b), the basic characteristic peaks of CQDs remain the same, indicating that the fundamental structures were mainly identical upon adding EP and do not show a profound influence. The broad peak at 3443 cm^−1^ was associated with the –OH stretching vibrations. The peak at 2924 cm^−1^ is ascribed to the stretching vibration of C–H, and the peak around 1632 cm^−1^ confirms the presence of carbonyl species (C=O). These results confirm the presence of hydroxyl and carboxylic functional groups.^31^ The peaks located at 1632 cm^−1^ and 1555 cm^−1^ confirm the presence of the CO–NH group, which indicates that the synthesized CQDs/SiO_2_ was amine-functionalized by APTES. The peaks at 1064 cm^−1^ and 796 cm^−1^ are attributable to the Si–O–Si stretching vibration and Si–O bending vibration, respectively.^32^ This indicates that the imprinted polymers incorporating CQDs@MIP have been successfully synthesized.

The phase structure and the crystalline nature of the synthesized silica-supported CQD nanoparticles were characterized by X-ray diffraction (XRD) analysis. For EP-leached CQDs/MIPs, a broad XRD peak can be observed, as shown in Fig. 4(c). The intense diffraction peak at 2*θ* = 23.5° corresponds to the (002) diffraction plane of the carbon material.^31^ The XRD pattern of unleached CQDs/MIPs is shown in Fig. 4(d). The sharp peaks around 2*θ* = 43.3° and 50.3° might be related to the presence of KCl and disappear after the washing process. It also reveals the amorphous structure in the carbon atoms affected by more oxygen-containing groups on the surface of the synthesized CQDs. In other words, most CQDs have very weak crystallinity.^33^ This amorphous structure relates to the surface functional groups of carbon dots, especially the SiO_2_ shell layer formed by APTES and TEOS.

**Fig. 5:**
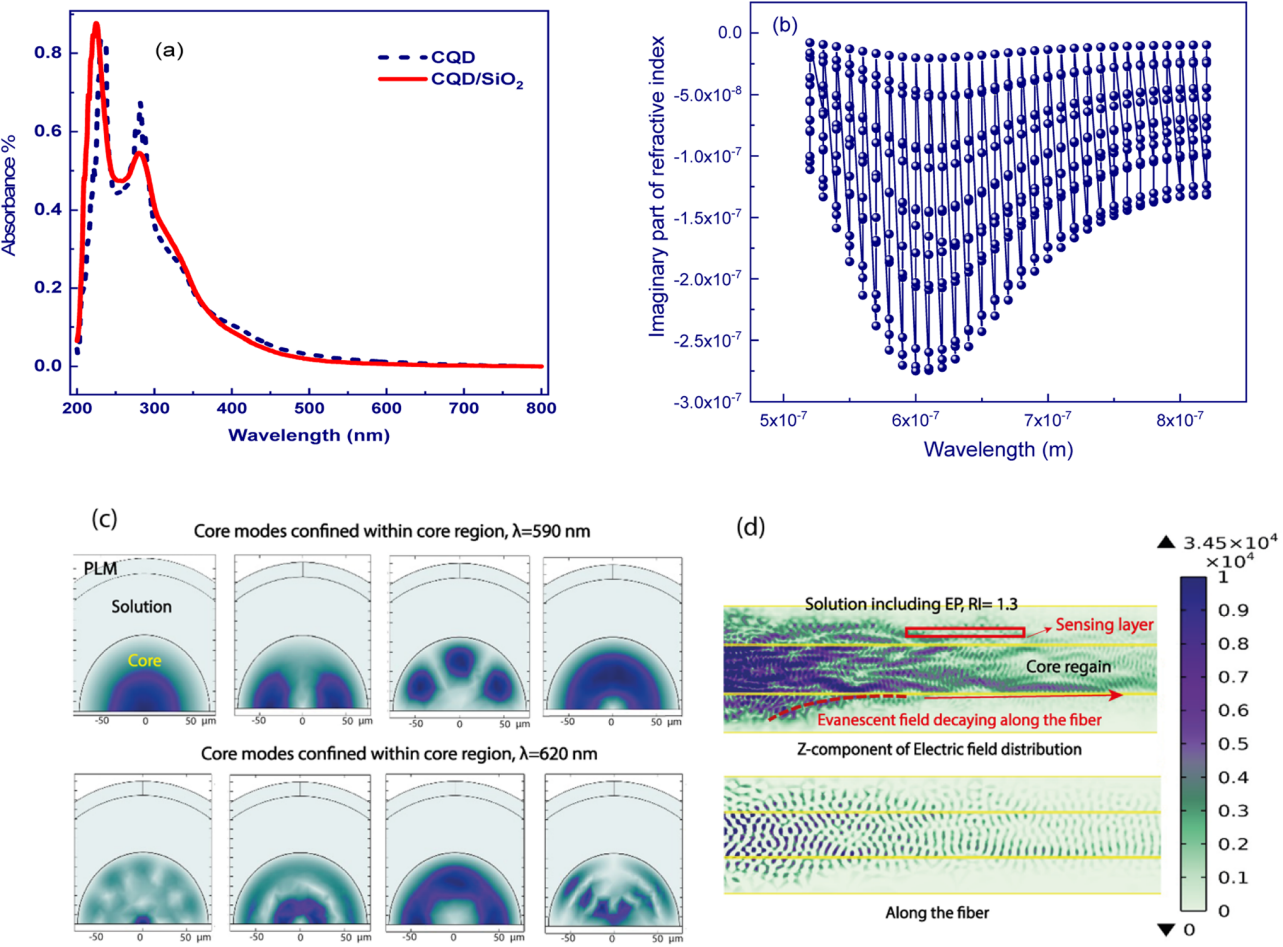
(a) UV-Vis spectra of CQDs/MIPs, and (b) the calculated imaginary part of the refractive index as the function of the wavelength. (c and d) Mode profile of the norm of *E* and the corresponding *z* components of core-guided modes of CQDs/MIPs coated on optical fibers, from both views of the cross-section and along the optical fiber.

It is well -known that manipulating the fiber cladding with appropriate coatings might lead to the LMR phenomenon, which is generated through attenuated total reflection. The real part of the refractive index is responsible for the LMR wavelength shift, whereas the LMR depth depends on the imaginary part.^34^ Specifically, LMR occurs when the real part of the coating permittivity (*ε* = *ε*′ + i*ε*′′) is positive and higher than its imaginary part and the material surrounding the coating. In terms of the coating refractive index *N*, 
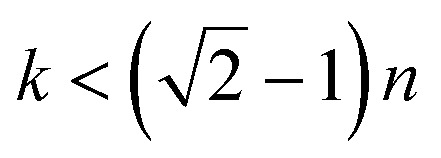
 and *n*^2^ − *k*^2^ > 1.45^2^, where *n* = Re[*N*] and *k* = Im[*N*].^35^ Therefore, we try to calculate the refractive index (*n*) based on the UV-Vis measured spectra of the synthesized detecting solution, which is similar to the CQD absorption spectrum as shown in Fig. 5(a), implying the decisive role of CQDs.

Herve and Vandamme^36^ suggested a relation between the refractive index and the energy gap in semiconductors based on light refraction and dispersion physics:1
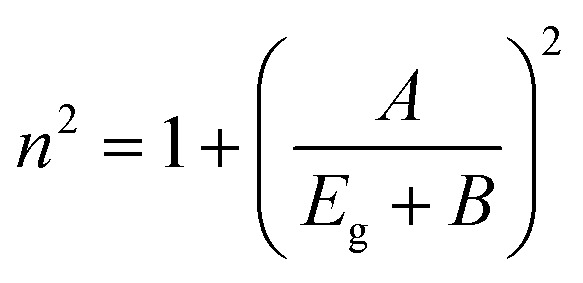
where *A* = 13.6 eV and *B* = 3.4 eV is a constant supposed to be the difference between the UV resonance energy and the band gap energy.^37^ The optical bandgap is calculated using the Tauc relationship and is shown in Fig. S2.†

The optical bandgap is obtained by extrapolating the straight line of |*hν*|^2^*versus* the photon energy plot to *ν* = 0. The original bandgap (*E*_g_) was calculated to be 3.98 eV, corresponding to the UV-Vis absorption (Fig. 5(a)). The value is approximately the same in both equations. The refractive index obtained from eqn (1) is 1.68. Also, the extinction coefficient is calculated using *k* = *αλ*/2π, in which the absorption coefficient *α* is calculated using Beer–Lambert's formula,^38^2
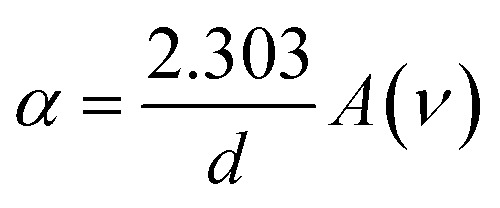
where *d* is the sample thickness and *A*(*ν*) is the absorbance. The extinction coefficient is roughly 1 × 10^−5^, which is nearly constant for the whole visible range. It can be realized that both real and imaginary parts of the refractive index are positive in the working wavelengths from 200–800 nm. Also, the real part is higher in magnitude than the imaginary part. This ensures the LMR occurrence, which is the main concept of this study. Herein, to disclose the concrete absorption sensing mechanism, the two-dimensional finite element method (2D-FEM) with commercial software (COMSOL Multiphysics) was used to analyze the effective refractive index and electric field distribution of the propagated modes in the core region. The main differential wave equation is solved within the whole structure considering the continuity and boundary conditions and more detailed theory and the main equation are reported in the Appendix of our earlier work^39^ for choosing a proper mesh distribution.

LMRs are generated when a guiding mode in the cladding experiences a transition to guidance in the coating. This causes a reorganization of the effective indices of the rest of the modes guided in the cladding simultaneously as their evanescent field is increased, increasing the transmission losses.^40^ So, in this calculation, the mode analysis technique is used for the fiber coating, and the normalized electric field is depicted in Fig. 5(c). The results supported for the first mode of this structure, which is acting as a waveguide, are presented in Fig. 5(c). To better understand the coupling of propagating light of some higher modes into the coating layer resulting in lossy modes, the imaginary part of the effective index for eleven modes was calculated *versus* wavelength and depicted in Fig. 5(b). It can be seen that the spectral region in which the imaginary value is more in magnitude is expected to be the lossy dip region of the experimental performance. Herein, the region from 580 to 620 nm with the obtained higher imaginary value introduces the resonance lossy mode wavelengths which are consistent with the following experimental results. To further clarify, the electric field core-guided mode distributions are illustrated at the propagated wavelength light of 590 nm in Fig. 5(c). It can be seen that the electric field distributions of the higher modes in the vicinity of the coated layer result in the strongest coupling between the core guiding modes and leaked modes of the coated layer. In this situation, the phases match well, and the energy of emerging higher order modes transfer to the coating layer, implying transit maximally into the lossy mode. The longitudinal point of view of the norm electric field and the corresponding *z* component electric field distribution of a guided core mode also is illustrated, as shown in Fig. 5(d). The decaying behavior of the evanescent field along the fiber longitudinal could be observed clearly such that the norm of the electric field and the *z* component in a magnified scale were confined in the interface facet, allowing the evanescent wave trapping in this region, leading to the sensing improvement. This ensures the LMR occurrence, which is the main concept of this study.

### 3.3 LMR-based sensing performance

In this section, the main results are reported, and the effect of the EP analyte and CQD concentration, as well as the dipping period on the sensitivity of the LMR-based optical fiber sensor is studied. In Fig. S4,† to experimentally analyze the LMR-based optical fiber sensor's performance, the transmission spectra are obtained for different CQD concentrations. The central wavelength related to the dip of the optical transmission spectrum is called the resonance wavelength, *λ*_res_ and the sensitivity of the sensor is defined as the ratio of the *λ*_res_ shift of the transmission to the changes in EP concentrations. In fact, the value of *λ*_res_ changes corresponding to the variations of the refractive index difference between the guiding modes in the fiber core and the leaky evanescent waves in the CQD/MIP coating surrounded by the sensing medium. This is mainly due to the fact that the refractive index is an ascending function of the EP concentration.

**Fig. 6:**
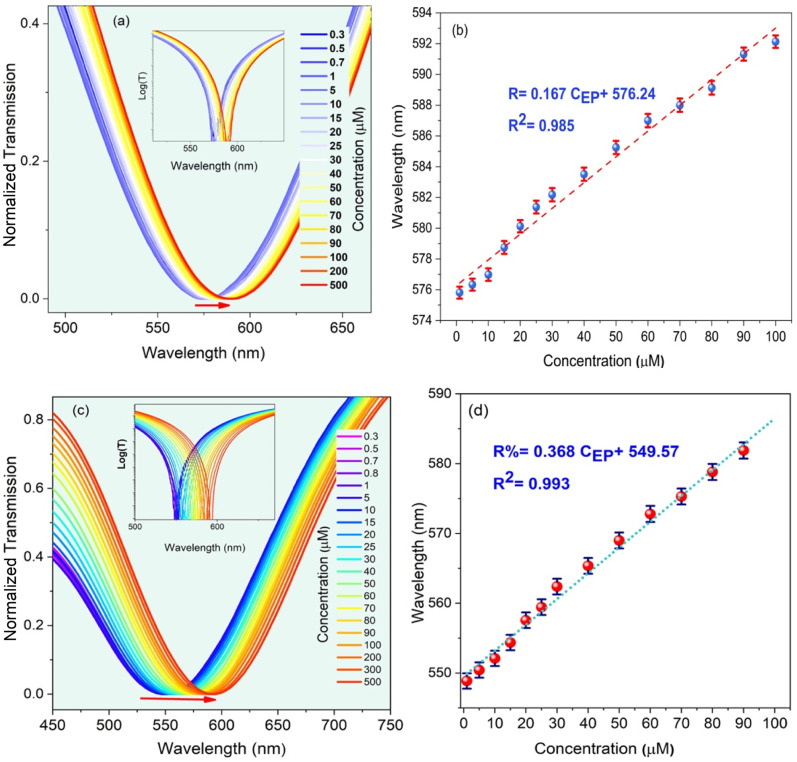
The LMR dip shift for different EP concentrations: (a and b) 7 mL and (c and d) 9 mL. In this case, the concentration of CQD solution was fixed at 15 mM.

In the next step, while keeping the CQD concentration at a constant value of 15 mL, the effect of EP concentration variations is studied, as shown in Fig. 6, which decides the density of target imprinted sites within the sensing region. The initial LMR wavelength is 567 nm, and it undergoes a red-shift to 597 nm when the EP concentration increases to 500 μM. In the same trend, as the EP concentration in the synthesized solution increases, a red-shift experienced by the LMR wavelength in the transmission spectra is observed. In order to clarify the concept of the lossy mode resonance and the corresponding shift, the logarithmic view is also illustrated in the inset of the transmitted spectra. As shown in Fig. 6(c and d), the optimal condition is for the case when CQD and EP concentrations are 15 mL and 9 mL, respectively. In this case, the sensitivity is reported to be 0.37 nm μM^−1^. A fitting curve of the data displays that the LMR wavelength shift is linear with a correlation factor of *R*^2^ = 0.99. It can be observed that the sensitivity behavior is essentially a smooth linear relationship, which is a vital requisite for practical sensors.

**Fig. 7:**
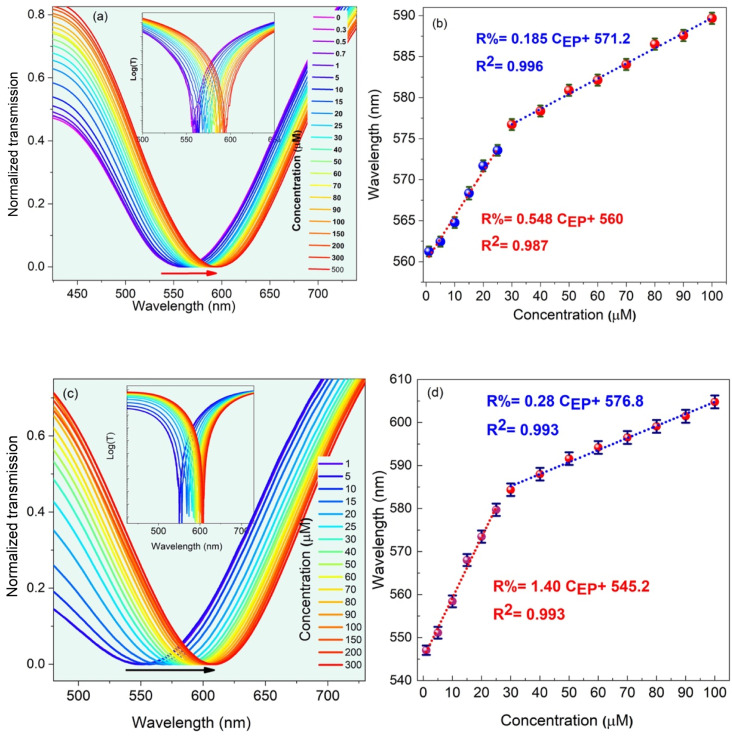
Effect of the elapsed time on the LMR shift, for (a and b) 3 h and (c and d) 7 h.

Finally, to evaluate the effect of the dipping period under the ultrasound irradiation on the optimum behavior of the LMR-based optical fiber sensor, the sensitivity is studied for the case when the molar concentration of CQDs and EP is 15 and 9 mL, respectively. At this stage, the optical fiber is immersed in the reaction solution, and the experimental data are collected at 3 h and 7 h and the results are shown in Fig. 7. Although experiments performed at 3 h and 7 h are capable of sensing EP, the sensor behavior at low and high EP molar concentration is still linear with different slopes (*i.e.*, different sensitivities). Fig. 7(b) suggests that the selective MIP leads to higher sensitivities at low concentrations. As the concentrations increase, the selective MIP cavities decrease due to the surface absorption, reducing the sensitivity. For some MIP thicknesses, the imprinting layer hinders the rapid electron transfer between CQDs and the EP analyte, causing sensitivity decline.^15^ Thus, the best result was obtained for an elapsed time of 5 h, with a sensitivity of 0.37 nm μM^−1^ and a correlation factor of *R*^2^ = 0.99, as shown in Fig. 7(c and d).

### 3.4 Selectivity of detection and applicability study

**Fig. 8:**
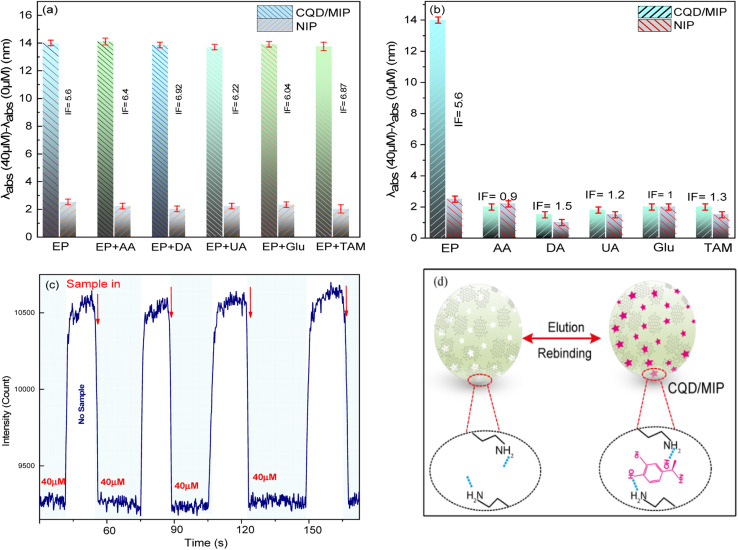
The selectivity responses of (a) CQD/MIP and CQD/NIP nanocomposites, to EP in the presence of different kinds of interferents (b) CQDs/MIPs to EP and different kinds of analytes, (c) repeatability, and stability plots of the as-prepared sensor probe with a 5 h sol–gel step in 15 mL of CQDs and 9 mL of EP and (d) the mechanism of specific binding of the target analyte at the MIP layer.

The selectivity of the LMR-based probe towards EP was analyzed by separately adding ascorbic acid (AA), dopamine (DA), urea (UA), glucose (Glu), and TAM (tamoxifen) to measure changes in the fluorescence intensity ratio. The fluorescence intensity ratios of NIPs and MIPs are presented under the optimized experimental conditions in Fig. 8. The results evidently demonstrate that the spectral selectivity of the template molecules for EP detection is significantly higher than that of the CQD/NIP nanocomposite. For this sensor, the imprinting factor (IF), which is selected to evaluate the materials selectivity, is calculated as IF = *R*_MIP_/*R*_NIP_.^41^ The calculated IF was 5.6, indicating that CQDs/MIPs possess highly selective recognition of EP. In other words, this relatively high IF reveals that the imprinting process can prominently increase the quenching efficiency and sensitivity. These results indicated that the CQDs/MIPs improve selectivity, sensitivity, and anti-interference abilities. Therefore, the as-prepared CQDs/MIPs can be used as a fluorescent probe to detect EP quantitatively. Meanwhile, the stability and selectivity experiments proved the high performance of the sensor system. Also, the fluorescence responses of CQDs/MIPs and CQDs/NIPs to various interferents in an aqueous solution were investigated to evaluate the practical effectiveness of our proposed LMR-based sensor for EP detection. The abnormal concentration of catecholamines (*e.g.* EP, DA and so on) can be related to a variety of diseases including Parkinsonism, Alzheimer’s disease, Schizophrenia, pheochromocytoma, various neuroblastoma and depression as well.^42^ Therefore, the effect of interferent compounds is investigated at different concentrations. The concentration of all the other interferents was 40 μM. As shown in Fig. 8(b), the CQDs/MIP response towards EP was higher than that of the CQDs/NIPs, which indicated selective adsorption of CQDs/MIPs for the template molecules. It is well-known that repeatability and stability are among the essential requirements for real-life sensors. To evaluate the practical applicability of the as-prepared sensor, initially, the output intensity was recorded in a sample without any analyte. Then, 50 μM sample was dispensed into the flow cell, and the intensity spectra were recorded again. The process was repeated for three cycles, and the sample was allowed to remain in the probe's vicinity in any measurement. The obtained results are shown in Fig. 8(c), where a negligible intensity change can be observed. Recent studies show that the CQDs have excellent service lives compared to the other QDs owing to the synergy of the bio-inspired structure and strong interactions between CQDs and MIPs.^6,43^ So, the CQDs could be used over prolonged periods of time. The device operation has been checked for up to four weeks, and the stability study showed an insignificant decrease in the shift compared to the initial response. The relative standard deviation (RSD) was calculated to be 0.16% for four independent measurements at the EP concentration of 40 μM. It can be concluded that the sensor offers acceptable stability (see ESI S5†). As illustrated in Fig. 8(d), CQDs/MIPs were manufactured using the surface-imprinted sol–gel technique. The reaction was conducted in the presence of APTES, TEOS, EP, and aqueous CQD solution. After this, the template molecule of EP was removed *via* methanol and acetic acid, leaving behind 3D cavities in the imprinting layer and a CQD/MIP sensor with selective binding sites fitting the shape of EP was obtained. They can efficiently bind related sites through hydrogen bonding of amino-functionalized groups within the sensing region and provide selective binding of target molecules.

### 3.5 Real sample analysis

To evaluate the applicability, the proposed LMR-based optical fiber sensor is utilized for the determination of the EP analyte in an injection solution and human urine. EP hydrochloride injection samples (standard content of EP 1 mg mL^−1^; 1 mL per injection) were purchased from a local pharmacy, and human urine samples were obtained from a local pathology clinic, Dr Yazdanpanah Medical Laboratory, in Yasouj city, Iran. For all cases, the real sample for the injection was approximately diluted to bring it to the concentration working range. Meanwhile, a standard stock solution of an EP equivalent to the real sample was prepared. The optical response of EP in the real sample solutions was estimated by adding different EP concentrations to the standard solution. Under the optimized conditions, three detection experiments were performed using the fabricated optical fiber sensor (*n* = 3). The corresponding analytical responses are recorded in Table 1S.† The recoveries obtained by the method for EP injection were in the range from 98.57 to 101%, with the RSD in the range of 0.25% to 0.62%. The data indicate that the results are in good agreement with the labeled amount of EP, which further suggests that the present CQD/MIP LMR-based sensor can effectively determine EP in pharmaceutical formulations and human urine samples.

Note that the limit of detection (LOD), which characterizes the detectable amount of near-zero analyte concentration, can be calculated based on the standard deviation of the response curve (*S*_y_) divided by the slope of the calibration curve (*S*) as LOD = 3.3(*S*_y_/*S*).^20^ In our analysis, to calculate the LOD, the optimized sensor probes with a 5 h sol–gel process, 15 mL of CQDs, and 9 mL of EP are preferred. It becomes as low as 72 μM. Table 1 provides a brief review and the obtained results of previously reported techniques for EP determination. Compared to the previously reported sensors, the CQD/MIP optical fiber sensor shows numerous advantages, such as good linearity and stability for EP sensing within a sufficiently wide range, low LOD, low cost, high sensitivity, and test rapidity.

**Table 1:** Comparison of various methods reported for EP determination

Method	Material	Linear concentration range (μM)	LOD (μM)	Reference
Electrochemical	Graphitic carbon nitride/N-doped carbon dot composite	1.0 × 10^−6^ to 1.0 × 10^−3^	3.0 × 10^−7^	44
Electrochemical	GQD–CS/CPE	360 to 38 × 10^4^	3.0 × 10^−4^	45
Optical sensor	Upconversion nanoparticles with different metal ions	(5–30) × 10^−3^	2.4 × 10^−3^	46
Electrochemical	MIP/graphene oxide	1 × 10^−2^ to 5.0 × 10^−1^	5 × 10^−3^	47
Electrochemical	*o*-PD–EP-MIP	0–100	Less than 13	48
Electrochemical	(MIP)/gold nanoparticles	9.0 × 10^−2^ to 1.0 × 10^2^	7.6 × 10^−2^	49
Electrochemical	Poly(DA)–nanogold/GCE	1–80	0.1	50
Optical fiber	MIP/carbon dot	1–100 μM	0.72 μM	This work

The main limitation is that the detection of EP in the picomolar range is difficult with ultra-level detection. Thus, long-wavelength biosensors may be needed in the future for the efficient detection of EP. Also, interferences between targets and probes may arise due to analyte-independent events such as light scattering by the sample matrix, excitation source variation, and concentration changes near the probe. In fact, ratio-metric measurements render a self-calibration for environmental interference and will efficiently overcome the limitations in the measurements. Ratio-metric fluorescent sensors detect intensity variations in fluorescence from two or more emission bands with distinct wavelengths, which is going to be considered for the future work of this group.

## 4. Conclusions

In summary, an LMR-based optical fiber sensor has been developed for the determination of epinephrine using CQD/MIP nanocomposites coated on the optical fiber cladding. To the best of our knowledge, this is the first experimental demonstration of the CQD-coated optical fiber sensor based on the LMR phenomenon. In our study, the structural and optical characterization of the as-synthesized CQDs was performed by HR-TEM, XRD, FT-IR, and UV-Vis spectroscopy analyses. Also, the mechanism of fluorescence emission was investigated. Then, the CQDs were employed in an optical fiber sensor after modification with MIPs and a direct ultrasonic irradiation technique. Experimentally, the absorptive LMR-based sensor is evaluated using a CQD/MIP solution and characterized in terms of the examination of the wavelength shift of the dip in the transmission spectrum. Under the optimal conditions, the sensitivity of the proposed LMR-based optical fiber sensor is reported to be 0.37 nm μM^−1^. So, sensitive detection of epinephrine at a low concentration can be guaranteed. Also, the LOD achieved is 0.72 μM, and the correlation coefficient is 0.99. The imprinting factor is 5.6, and good recoveries up to 102.5% are attained in the detection experiment of EP in aqueous samples. The sensitivity behaviour is essentially a smooth linear relationship, which is a vital requisite for practical sensors. The findings indicated that CQDs/MIPs are ideal sensors for the rapid and accurate determination of EP in real aqueous samples. So, the developed optical fiber sensor provides another approach for chemical process monitoring, biomedical and industrial applications.

## Conflicts of interest

The authors declare no competing interests.

## Supplementary Material

NA-005-D2NA00687A-s001
