# Supplementary material for: Smart green CQD@SiO2 hybrid coated optical fiber manifesting dual versatile absorptive and MIP features towards epinephrine detection
Source: Nanoscale Adv. 2022 Dec 19;5(2):459–70. doi: 10.1039/d2na00687a (PMC9846438; doi:10.1039/d2na00687a)
Supplement: NA-005-D2NA00687A-s001 [file NA-005-D2NA00687A-s001.pdf]

Electronic Supplementary Material (ESI) for Nanoscale Advances.
This journal is © The Royal Society of Chemistry 2022

Smart Green CQD@SiO_2 hybrid Coated Optical Fiber Manifesting Dual Versatile Absorptive and MIP features towards Epinephrine Detection

Tahereh Azargoshasb [1], Roghaieh Parvizi [2], Forough Bozorgzadeh [3], H. Ali Navid [1], and Hadi Heidari [4]

[1] Department of Laser and Optical Engineering, University of Bonab, Bonab 5551761167, Iran.
[2] Department of Physics, College of Sciences, Yasouj University, Yasouj 75914-353, Iran.
[3] Physics Department, College of Sciences, Shiraz University, Shiraz, Iran.
[4] James Watt School of Engineering, University of Glasgow, Glasgow, G12 8QQ, United Kingdom.

Supporting Information

S1: EDX spectrum of carbon dot nanoparticles.

S2: Tauc plot.

S3: Raman spectrum of the synthesized CQDs.

S4: Transmission spectra

S5: Stability plots.

S6: HR-TEM images of CQDs

1. Chemicals

Epinephrine (EP), Tetraethyl orthosilicate (TEOS), and (3-Aminopropyl) Triethoxysilane (APTES, 97%), hydrochloric acid (HCl), sulfuric acid (H_2SO_4), hydrogen peroxide (H_2O_2), hydrofluoric acid (HF 38-40%) and Glycine were obtained from Merck Co. Plastic-cladding silica multi-mode fibers (PCS-MMFs) of 400/425 μm core/cladding diameter (FT400EMT) were provided by Thorlabs Inc. Cabbage, as a natural carbon source, was purchased from a local grocery store.

2.Instrument for characterization

Fourier transform infrared (FT-IR) spectra were recorded using Thermo Scientific Nicolet iS10 spectrometer, and the UV-VIS spectroscopy was carried out on a Mettler Toledo UV/Vis spectrophotometer UV7. Fluorescence spectra were obtained using Cary Eclipse fluorescence spectrophotometer (Agilent Technologies). High-resolution transmission electron micrographs (HR-TEM) were carried out on a transmission electron microscope (Tecnai G2 F20, Philips Healthcare) operated at an accelerating voltage of 200 kV. The X-ray diffraction (XRD) pattern of the CDs was recorded using an X-Ray diffractometer (Philips PW 1730/10). Field emission scanning electron microscopy (FE-SEM) image was applied on SEM FEI Quanta 200, equipped with an energy-dispersive X-ray analyzer (EDX) to investigate the morphology and elemental composition. Raman spectra were recorded on a Raman microscope (Renishaw inVia). The experiments were performed in an ultrasonic bath at 20 kHz in a continuous cycle for 30 min.

3.optical fiber etching and preparation

The Multimode Fiber (TECS-hard-clad multimode optical fiber, 400 μm diameter Core, 0.39 NA, Step Index) sensing region was realized by chemically removing the clad of a certain length (~12 mm) at the middle part of the fiber, followed by a simple procedure of etching the fiber core using hydrofluoric acid, and the etching period was optimized as explained in our earlier works[1]. Then, the fiber sample was washed with doubly distilled water and dipped into freshly prepared piranha solution (3:1 ratio of concentrated sulfuric acid (H2SO4) and 15% hydrogen peroxide (H2O2)) for 8 h to clean organic impurities

from the fiber core. Thus, a hydroxylated surface is prepared for the subsequent interaction with the silica coupling agent, i.e., APTES. Following this, the stripped fiber was rinsed thoroughly with deionized water and dried completely.

S1: EDX spectrum of CQD/MIP and carbon dot nanoparticles

The EDX elemental mapping image of spinach-CQD nano-composite is shown in Figure S1 of the supporting information, demonstrating that the surface-functionalized CQDs were successfully produced. The elemental mapping of spinach-CQD nano-composite (CQD/MIP) is shown in Figure S1, identifying elements such as carbon (C), Chlorine (Cl), nitrogen (N), oxygen (O), and silicon (Si). The nitrogen (N) presence reveals that APTES is well-functionalized on the fiber surface. Also, the table shows the mass parentage that indicates the spinach-CDs formed from Si (69.4%) and O (17.0%).

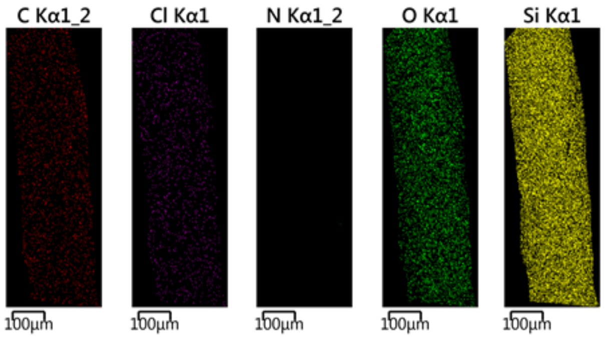

(b): table showing the mass parentage of the elements in Spinach-CQD

Element	Weight %	Atomic %	Uncert. %	k-Factor	Absorption Correction
**C (K)**	19.28	26.87	0.14	1.885	0.949
**O (K)**	59.64	62.42	0.49	6.926	0.958
**Na (K)**	4.13	3.00	0.05	1.293	0.976
**Mg (K)**	0.11	0.08	0.00	1.093	0.984
**P (K)**	0.36	0.19	0.01	1.002	0.995
**S (K)**	3.58	1.87	0.04	0.966	0.996
**Cl (K)**	0.47	0.22	0.01	1.012	0.997
**K (K)**	12.38	5.30	0.07	1.040	0.998

Figure S1: (a) the elemental mapping of CQD/MIPs displaying: carbon (C), Chlorine (Cl), nitrogen (N), oxygen (O), silicon (Si), and (b) the table showing the mass parentage of the elements in spinach-CQD.

S2: Tauc plot

The optical bandgap is calculated using the Tauc relationship and is shown in Fig. S2. The indirect bandgap is obtained by extrapolating the straight line of $|h\upsilon|^2$ versus photon energy plot to $\upsilon = 0$. The originated bandgap (E_g) was calculated to be $3.98\ eV$, corresponding to the UV-Vis absorption (Fig. S2).

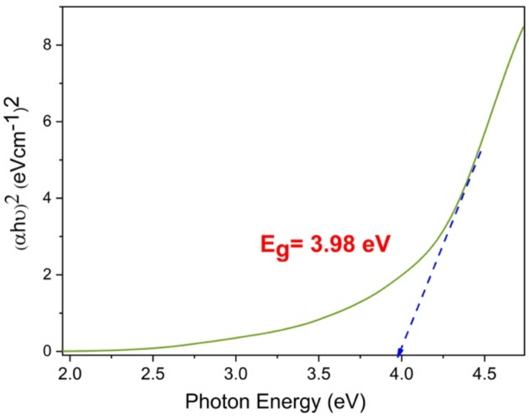

Fig. S2 Tauc plot for determination of indirect bandgap.

S3. Raman spectrum of the synthesized CQDs

The Raman technique is also a non-destructive robust tool for characterizing of CQDs. Fig. S3 displays the Raman spectrum of the synthesized CQDs nano-composite. The D band was identified at 1389 cm^{-1} as sp^3 hybridized vibrations, representing the disorder degree of the material structure, and the G band due to sp^2 hybridized vibrations was found at 1414 cm^{-1}, which was caused by the well-structured CQDs. The disorder degree can be determined using the Raman intensity ratio, calculated using the formula I_D/I_G. In our present work, the I_D/I_G ratio is 1.01, which shows that CQDs are amorphous in structure. This finding is consistent with the earlier XRD analysis (see the discussion in Figure 4).

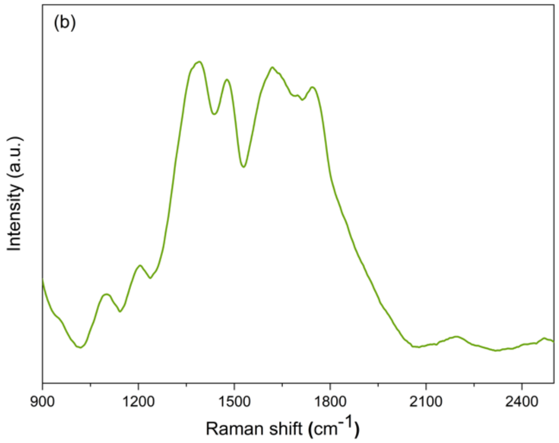

Fig. S3 Raman spectrum of the synthesized CDs.

S4: Transmission spectra

To evaluate the effect of the concentration of CQDs/MIPs on the sensitivity response of the sensor toward EP, the transmission spectra of different concentrations of CQDs were obtained at a fixed dipping period of 6 hr. It is found that depending on the concentrations of CQDs, the LMR wavelength can be fine-tuned, which is an advantage of using the CQDs. To optimize the working concentration of CQDs/MIPs nanocomposites, the optical transmission spectra for different CQD concentrations of 5 mL, 10mL, and 15 mL are obtained and shown in Figs S4(c), S4 (a)- S4 (b), respectively. Also, the LMR wavelength variations with the CQD concentration are displayed in Figs S4 (a)-(b). It can be observed that for the case that CQD molar concentration is fixed at 10 mL, the sensitivity at low (high) concentrations in the linear regime equals 0.223 nm/μM (0.094 nm/μM). Also, for the CQD molar concentration of 15 mL, sensitivity increases from 0.39 nm/μM at low concentrations to 0.18 nm/μM at high concentrations. It can be concluded that with a fixed CQD concentration, the sensitivity is decreased for the higher range of the target molecule of EPs' concentration. This might be due to the memory effect of target molecule measurement during successive analyzing tests. Also, it can be concluded that λ_{res} red-shift against the concentration of EP is attributed to the increased effective refractive index of the sensing layer. In fact, more surface defects, rather surface oxidation or other effective modification, leads to a red-shifted emission [2]. Indeed, the lossy behavior originates from the polymerization of TEOS and APTES on the surface of CQDs and the presence of charges related to the Si-O-groups, hydroxyl ions, or ammonium ions near the CQDs' surface that introduce a versatile lossy sensing layer [2]. Following the characteristic dip shown by the red arrow in the inset of Fig. S4 (b), a higher CQDs concentration can lead to more absorptive specification, leading to a more pronounced red-shift in the transmission spectra. The optimal response was obtained when CQDs/MIPs concentration was 15 mL. So, 15 mL CQDs/MIPs were used in further experiments.

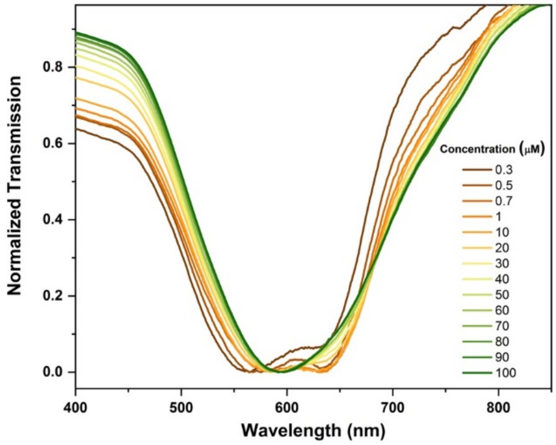

Fig. S4 (c)Transmission spectra of the LMR-based optical fiber sensor, for CQD concentration of 5mL

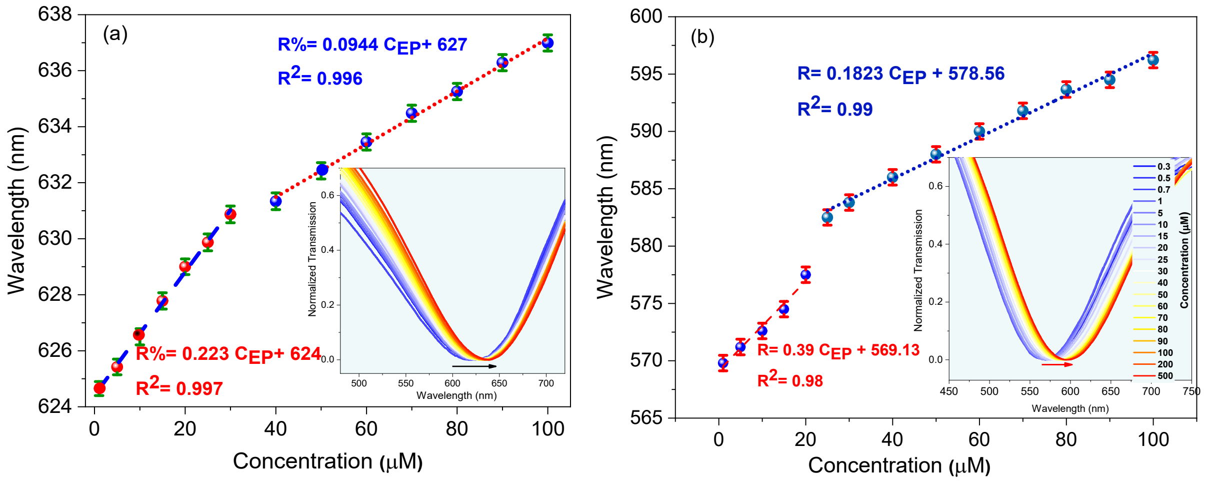

Fig. S4 (a) LMR wavelength versus EP concentration with the inset of transmission spectra of the LMR-based optical fiber sensor in 10 mL CQD, (b) Transmission spectra of the LMR-based optical fiber sensor and LMR wavelength versus EP concentration in 15 mL CQD.

S5: Stability plots

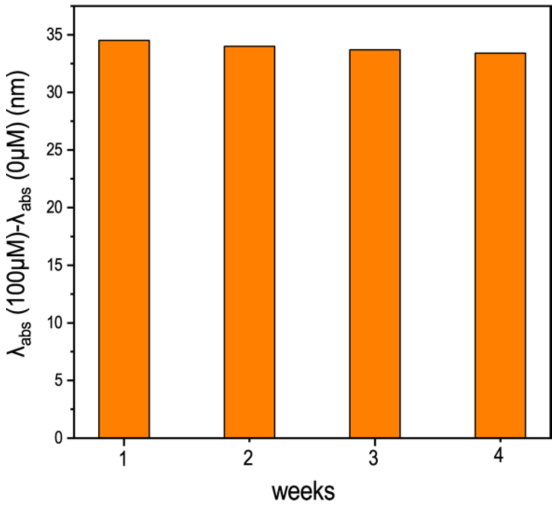

Fig. S5 stability plots of the as-prepared sensor probe with 5 h sol-gel step in 15 mL CQD and 9 mL EP.

Table 1S. Detection results of EP sensing in human urine and injection samples.

Sample	Add (μM)	Found (μM)	Recovery %	Relative standard deviation (RSD) (%, n=3)
EP injection (10μM)	40	50.5	101	0.62
	50	60	100	0.48
	60	69	98.57	0.25
Human urine	0	ND	-	
	20	19.5	97.5	0.73
	30	29.5	98.33	0.34
	40	41	102.5	0.58

S6: HR-TEM images of CQDs

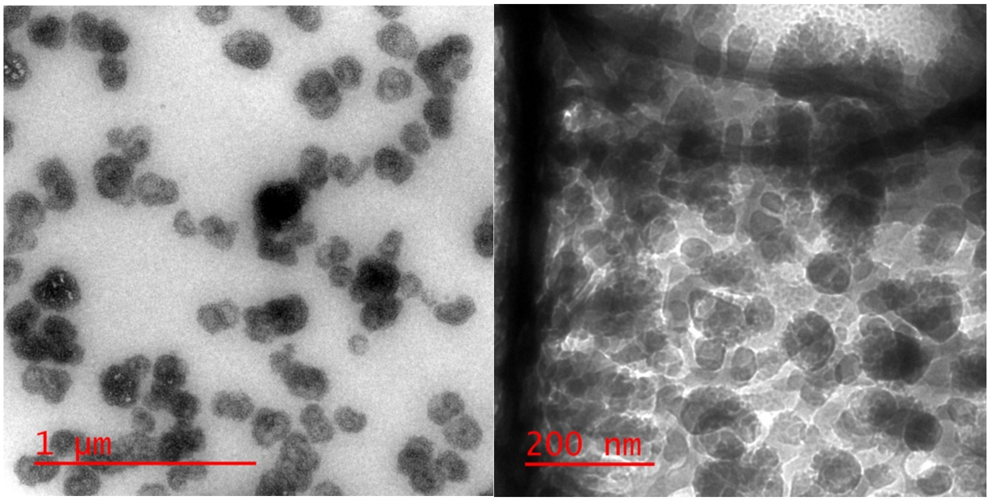

[1] T. Azargoshasb, H. A. Navid, R. Parvizi, and H. Heidari, "Evanescent wave optical trapping and sensing on polymer optical fibers for ultra-trace detection of glucose," *ACS omega,* vol. 5, no. 35, pp. 22046-22056, 2020.
[2] X. Sun and Y. Lei, "Fluorescent carbon dots and their sensing applications," *TrAC Trends in Analytical Chemistry,* vol. 89, pp. 163-180, 2017.
